# Reading performance in Portuguese children from second to tenth grade with the MNREAD reading acuity test

**DOI:** 10.1016/j.optom.2023.05.003

**Published:** 2023-06-14

**Authors:** Karthikeyan Baskaran, Aurélie Calabrèse, Laura Hernandez-Moreno, Diana Santos, Antonio Filipe Macedo

**Affiliations:** a Department of Medicine and Optometry, Linnaeus University, Kalmar, Sweden; b Aix-Marseille University, Marseille, France; c Laboratoire de Psychologie Cognitive, CNRS, Marseille, France; d Low Vision and Visual Rehabilitation Lab, Department and Center of Physics—Optometry and Vision Science, University of Minho Braga, Braga, Portugal

**Keywords:** Children, Reading performance, MNREAD acuity chart, Schoolchildren

## Abstract

**Purpose:**

To assess reading performance and report normative values for normal sighted Portuguese schoolchildren using the Portuguese version of the MNREAD reading acuity chart.

**Methods:**

Children in the 2nd, 4th, 6th, 8th^,^ and 10th grade in Portugal were recruited for this study. One hundred and sixty-seven children from 7 to 16 years of age participated. The Portuguese version of the printed MNREAD reading acuity chart was used to measure reading performance in these children. The non-linear mixed effects model with negative exponential decay function was used to compute maximum reading speed (MRS) and critical print size (CPS) automatically. Reading acuity (RA) and reading accessibility index (ACC) were computed manually.

**Results:**

The mean MRS in words-per-minute (wpm) for the 2nd grade was 55 wpm (SD = 11.2 wpm), 104 wpm (SD = 27.9) for the 4th grade, 149 wpm (SD = 22.5) for 6th grade, 172 wpm (SD = 24.6) for 8th grade and 180 wpm for the 10th grade (SD = 16.8). There was a significant difference in MRS between school grades (*p* < 0.001). Participants’ reading speed increased by 14.5 wpm (95% CL: 13.1–15.9) with each year of increase in age. We found a significant difference between RA and school grades, but not for CPS.

**Conclusions:**

This study provides normative reading performance values for the Portuguese version of the MNREAD chart. The MRS increased with increasing age and school grade, while RA shows initial improvement from early school years and gradually stabilizes in the more mature children. Normative values for the MNREAD test can now be used to determine reading difficulties or slow reading speed in, for example, children with impaired vision.

## Introduction

Reading is a complex task that involves visual sensory input, accurate fixational eye movements^1^^,^^2^ and high level cognitive aspects of comprehension.^3^ Assessment of reading performance can provide guidance for prescription of reading aids in low vision patients.^4^ Therefore, it is important that there are normative values available for various age groups for commonly used tests according to the language. The MNREAD acuity chart is a reading test that has been extensively used to determine reading performance in both children and adults with normal or with impaired vision. The MNREAD acuity chart was developed by Legge and colleagues in English ^5^ and has been widely adopted in many languages such as Portuguese,^6^^,^^7^ Spanish, French, Italian,^8^ German, Dutch, Greek,^9^ Turkish,^10^ Hindi, Korean and Japanese.

The Portuguese version of the MNREAD chart has been built with the participation of adults and children.^6^ Authors reported that the 38 sentences included in the test were read at between 95 and 125 words per minute (wpm) among children in the third grade in Brazil. However, the paper lacks normal values for the third grade or reading performance in children across a wide range of ages or school years. While normative data for reading performance in normally sighted children is available for various age groups in English ^11^ and Italian,^8^ there is no such data for the Portuguese version of the MNREAD test.

The availability of normative values for reading performance for a test like the MNREAD is crucial for many reasons. For example, normative values can help with screening of reading disabilities in children with normal vision, such as dyslexia. Although, probably more important, knowing the expected reading speed for different age groups can help in optimizing magnification for children with vision impairment. Optimal magnification should allow visually impaired children achieving their maximum reading potential. To know how compromised reading speed is by vision impairment is necessary to have normative values from children with normal sight. Our purpose was to assess reading performance and report normative values in normally sighted Portuguese schoolchildren using the Portuguese version of the MNREAD reading test.

## Methods

### Participants

Children in the 2nd, 4th, 6th, 8th, and 10th grade from Portugal were recruited to participate in this study. Children and parents (or legal guardians) were informed about the purpose of the study and signed an informed consent before the child's participation in the study. The study was conducted in accordance with the tenets of the Declaration of Helsinki, reviewed and approved by the ethics committee for Life Sciences and Health of the University of Minho (SECVS 147/2016).

A total of 167 children from 7 to 16 years of age participated, the mean age was 12 years (SD = 3 years) and 48% were females. We chose to include specific school grades starting with the second grade due to several reasons: 1) we started with children in the 2nd grade because those in the 1st grade were expected only to do word naming, not fluent reading, at the time of data collection, 2) we opted to measure reading speed every second year to test if changes were occurring as expected in line with cognitive development. Measuring every successive grade would have been unfeasible and would have provided minimal or no additional information to address the research question of the current study, 3) we stopped in the 10th grade under the assumption that reading speed would have by then reached a plateau and be adult like.

All children in the class were invited to participate and, for ethical and inclusion reasons, reading was measured in all those children that volunteered. However, for this report only those fulfilling the inclusion criteria were analyzed. The criteria were: 1) distance visual acuity 0.1 logMAR in either eye with the presenting correction and/or with the pinhole measured in a dimly lit room assessed with the ETDRS chart, 2) no strabismus or binocular vision problems assessed qualitatively by cover-test, or any externally visible signs of eye disease, 3) no self-reported or parent-reported eye diseases and 4) no learning difficulties such as dyslexia or ADHD reported by teachers.

Visual acuity was assessed using an internally illuminated high contrast ETDRS chart, model 2425E, luminance180 cd × m^2^ (https://www.precision-vision.com/) at 4 m.^12^ Visual acuity was measured using the ETDRS-Fast procedure where the children were asked to identify only one letter per line briefly pointed by the examiner. At the first letter that is not correctly identified (usually closer to the threshold) the child was asked to read the entire preceding row. From this point onwards the fast procedure is identical to standard procedure asking the child to read all the letters in each row until it has become evident that no meaningful reading can be obtained.^13^ The errors were documented in a score sheet and the visual acuity was calculated by scoring each correctly read letter as 0.02 logMAR.

In cases in which the tested visual acuity was lower than the required value of 0.1 logMAR, we used a pinhole to determine if acuity would improve. When acuity improved with the pinhole it was assumed the cause of reduced distance acuity was poor refractive error correction.

### MNREAD data collection and extraction

**Fig. 1:**
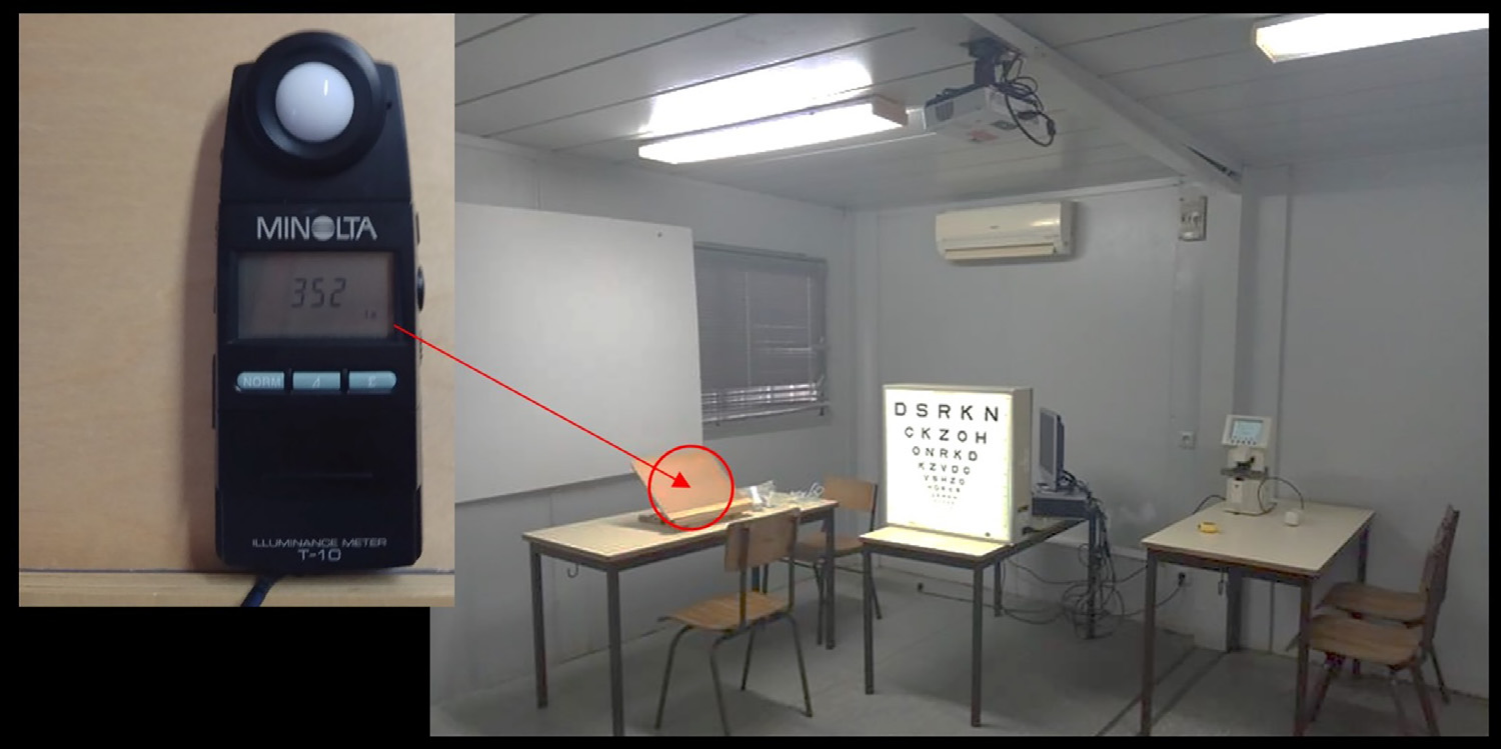
The right image shows the experimental setup of the room used for obtaining measurements using the MNREAD chart. The natural light was avoided by pulling down the blinds, the chair where participants sat to measure acuity is not in the picture. The table, chair and reading tray used for the MNREAD test is visible highlighted by the circle. In the magnified left image highlighted by the arrow and a circle shows the luxmeter with an illuminance (Il) reading of 352 lx that can be converted to luminance (*L*) using the formula: *L* = Il × reflectance/pi. Assuming a reflectance of 95% for the white background of the MNREAD test, the luminance in the test would be 106 cd/m^−2^. The focimeter that was used to measure the habitual spectacle correction is also visible in the picture.

The Portuguese version of the printed MNREAD chart was used to measure reading performance.^6^ The room settings are shown in Fig. 1. Reading distance was set at 40 cm and the participants read the chart binocularly. With their habitual correction, if any, children were instructed to read the sentences aloud as fast and accurately as possible from the largest to the smallest print size. The reading time and number of misread words were recorded on a score sheet by the experimenter (LHM) who has extensive experience in performing the test.^4^^,^^7^^,^^12^ Children had a practice run to get familiar with the task using one of the 2 versions of the charts available, the chart version for the training was randomly selected, the alternative version was used for measuring the reading parameters reported here. Measurements were performed during the normal school hours, typically any time between 8:30 and 15:30.

Data that was collected on the scoresheet was then processed in *R*.^14^ The mnread-R package was used to plot individual MNREAD curves of reading speed as a function of print size.^15^ The NLME model with negative exponential decay function calculated the maximum reading speed (MRS) and critical print size (CPS) automatically. CPS was calculated at 80% MRS with NLME model, as this cut-off value was found to be reliable in previous studies.^7^^,^^16^ Reading acuity (RA) and reading accessibility index was calculated manually. The standard formula for calculating reading acuity was 1.4 − (sentences × 0.1) + (errors × 0.01) and for reading accessibility index (ACC) was mean reading speed for the largest print sizes on the chart divided by 200.^17^

## Data analysis

A Shapiro–Wilk's test (*p* > 0.05) and a visual inspection of the histograms, normal *Q*–*Q* plots and box plots showed that only MRS was normally distributed. A one-way ANOVA with post hoc Tukey was conducted to investigate the effect of school grade on MRS. We also investigated the effect of age on MRS by performing a linear regression and quadratic fit. A non-parametric Kruskal–Wallis test with post hoc multiple comparison was performed for CPS and RA. Statistically analysis was performed using SPSS (IBM-SPSS v26, Illinois) and the graphs were plotted using GraphPad Prism (GraphPad, CA).

## Results

### Spectacle correction

The mean and median distance visual acuity for the 167 participating children for the right eye was −0.02 logMAR (S.D. = 0.13), −0.04 logMAR (IQR = 0.14) and for the left eye was −0.03 logMAR (S.D. = 0.12), −0.02 logMAR (IQR = 0.12). Table 1 summarizes, for each grade, the number of children who either had no correction or presented spectacle correction. The power of the spectacles was measured using a focimeter and categorized by type of refractive error. All children who had refractive correction had similar correction in both eyes except for one child in 10th grade who had an anisometropia (RE: +1.00 DS/LE: +5.00DS/−5.75 DC × 170°) but with equal vision of 0.0 logMAR in both eyes.

**Table 1:** Summary of the number of children in each grade along with the type of spectacle correction they had during the reading test.

Grade	*N*	Spectacle correction
2nd	26	1 hyperopic astigmatism 25 no spectacles
4th	34	1 astigmatic 3 hyperopic/hyperopic astigmatism 30 no spectacles
6th	39	7 myopic/myopic astigmatism 8 hyperopic/hyperopic astigmatism 24 no spectacles
8th	34	3 myopic/myopic astigmatism 3 hyperopic/hyperopic astigmatism 2 astigmatic 26 no spectacles
10th	34	5 myopic/myopic astigmatism 2 hyperopic/hyperopic astigmatism 1 astigmatic 26 no spectacles

**Fig. 2:**
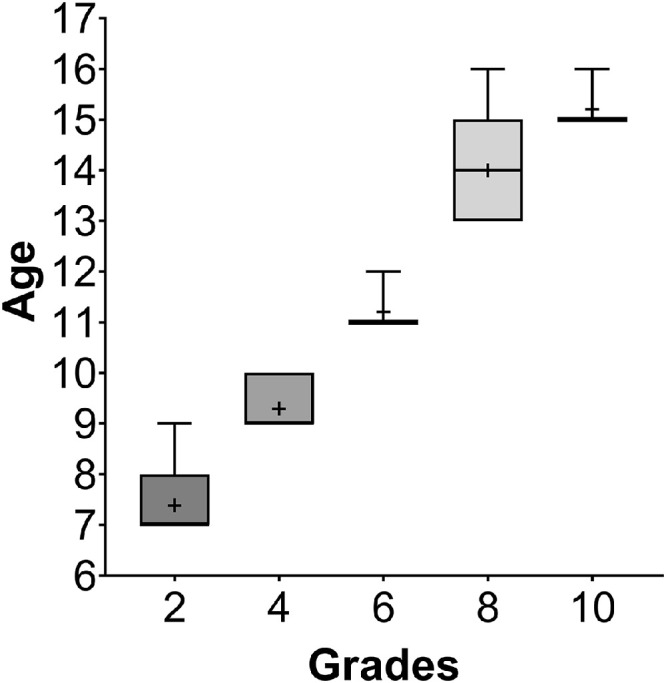
A box and whisker graph showing the age distribution at each grade. Boxes show 25th to 75th percentile and whiskers range from 5th to 95th percentile and the horizontal line represents the median and “+” symbol denotes the mean.

Fig. 2 shows the median age of the students at each grade.

### Maximum reading speed

**Fig. 3:**
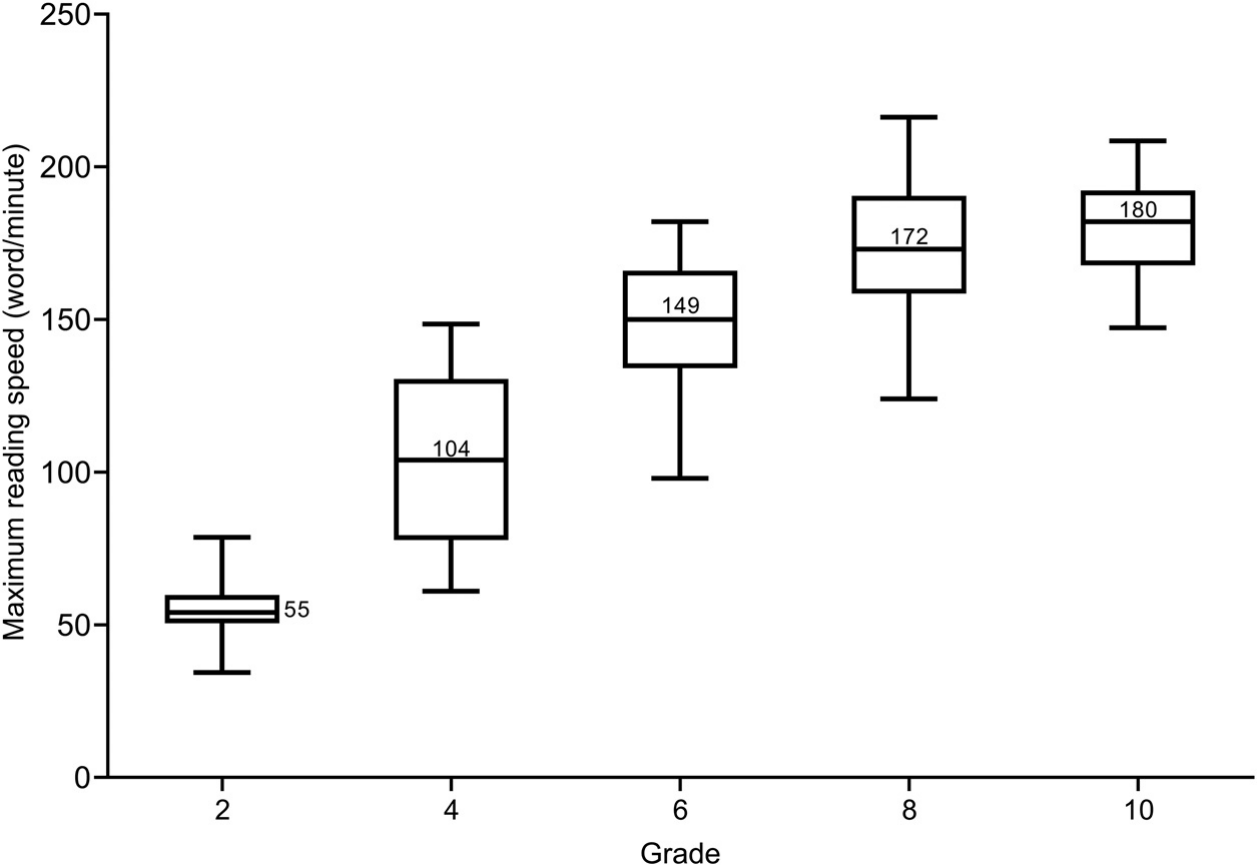
Box and whisker plot showing the MRS for each school grade. Boxes show 25th to 75th percentile and whiskers range from 5th to 95th percentile, the number inside the box sign indicates the mean reading speed in wpm (except for 2nd grade where it is located on the right side of the box), and the horizontal line represents the median.

Fig. 3 shows the mean MRS given in wpm for all grades. The mean MRS for the 2nd grade was 55.2 wpm (SD = 11.2), 4th grade was 103.7 wpm (SD = 27.9), 6th grade was 148.5 wpm (SD = 22.5), 8th grade was 171.9 wpm (SD = 24.6) and 10th grade was 180.4 wpm (SD = 16.8). One-way ANOVA confirmed statistically significant differences in MRS between grades, *F*(4, 162) = 168.0, MSE= 479.0 (*p* < 0.001). A post-hoc Tukey multiple comparison showed that MRS increased significantly from the 2nd to 8th grade (*p* < 0.001) but the difference between participants in the 8th and 10th grade was not statistically significant (*p* = 0.141).

The mean reading accessibility index (ACC) for the 2nd grade was 0.26, 4th grade was 0.51, 6th grade was 0.74, 8th grade was 0.85 and 10th grade was 0.92.

**Fig. 4:**
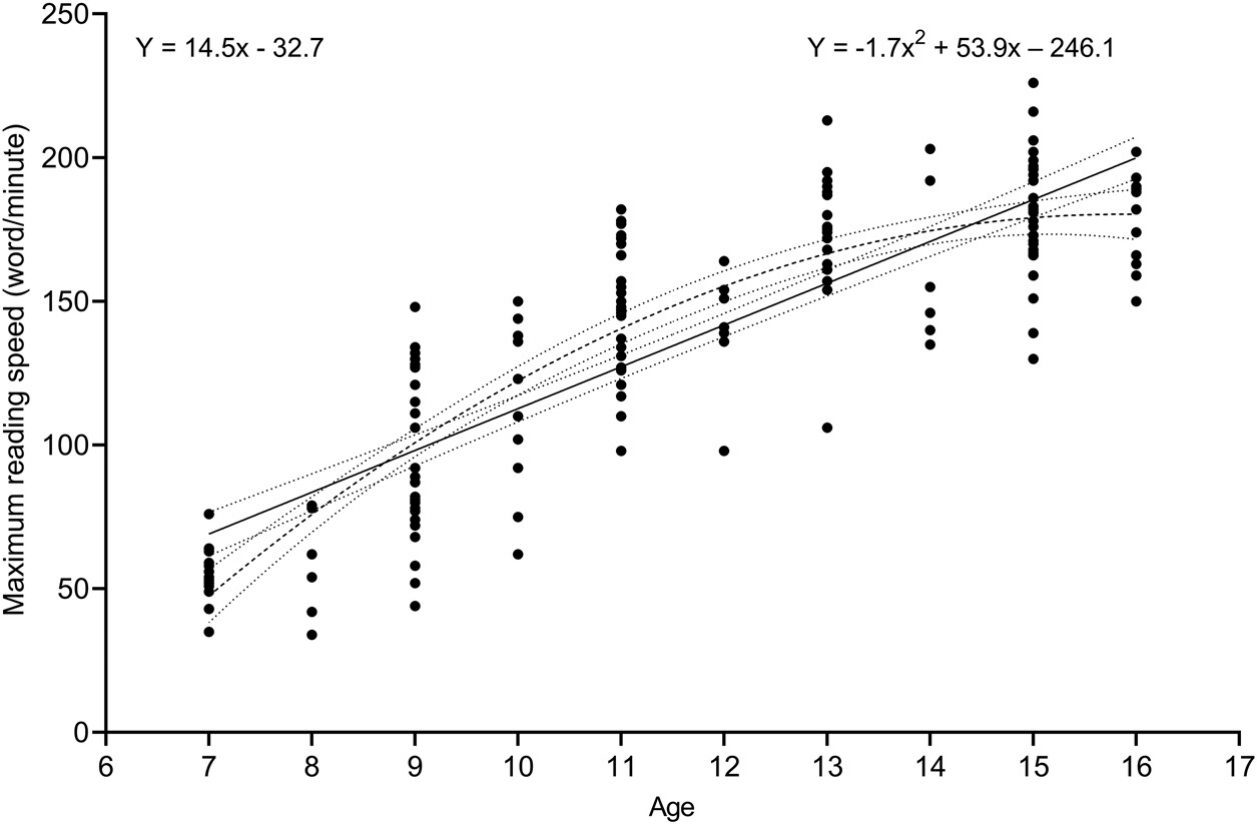
Scatter plot depicting a significant increase in reading speed with increasing age. The straight line traces the linear fit, with *R*^2^ = 0.722 and curved broken line showing the quadratic fit, with *R*^2^ = 0.774 with dotted lines representing the 95% confidence limits for both fits.

A simple linear regression was calculated to predict MRS based on age. A significant regression equation was found *F*(1, 165) = 429.00 (*p* < 0.001), with an *R*^2^ of 0.72. Linear regression revealed that participants’ reading speed increased by 14.5 wpm (95% CL: 13.1–15.9) with each year of increase in age. A second-order quadratic fit was also calculated with *R*^2^ of 0.77 and the equation for the fit was *Y* = −1.7*x*2 + 53.9*x* − 246.1. The superior quadratic fit shows that after the age of approximately 14 years, reading speed for the MNRAED test reaches a plateau. Fig. 4 shows a linear and quadratic fit for MRS data across age.

**Fig. 5:**
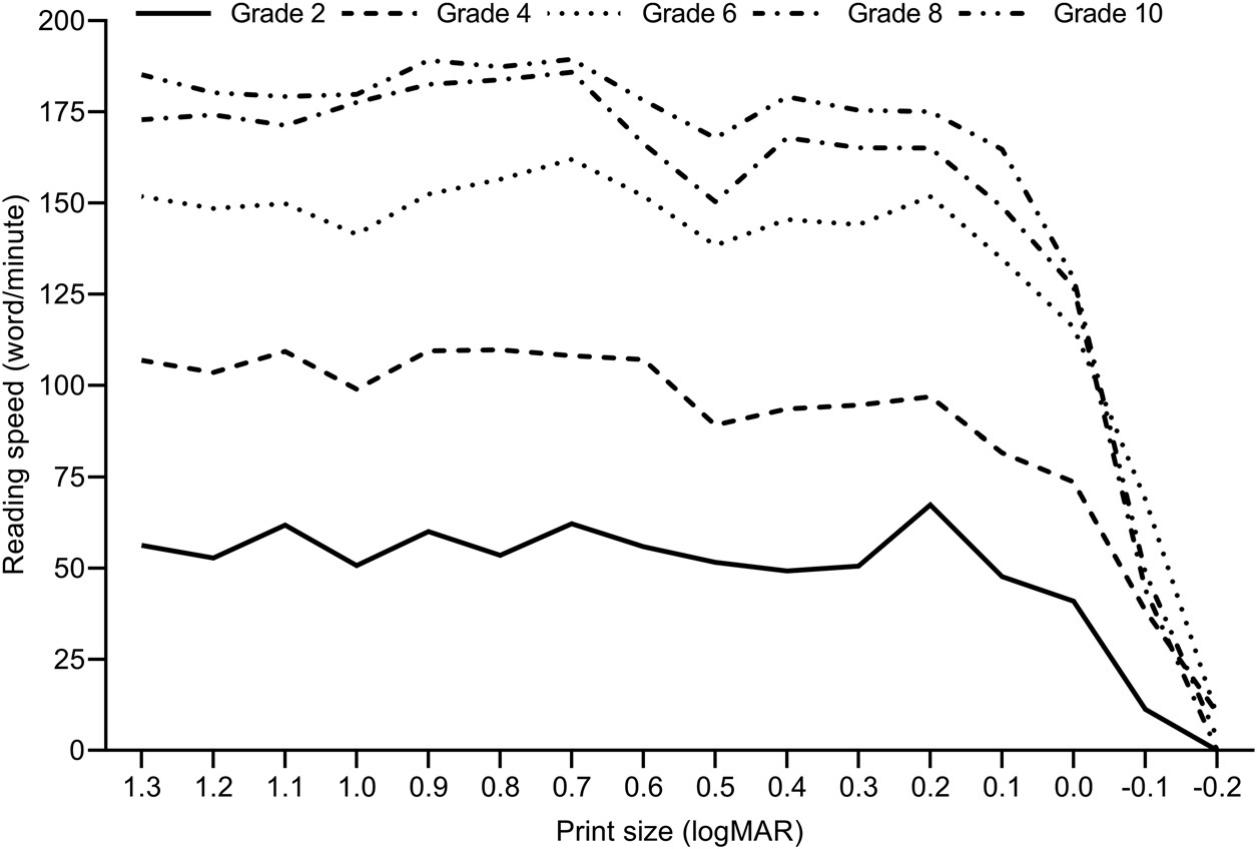
Mean reading speed for all sentences in the MNREAD chart, across five groups of children in the same grade.

Fig. 5 shows mean MRS for each print size, across five grades. The reading speed was relatively stable across 12 print sizes (1.3 to 0.2 logMAR) and reduced, as per definition, after the CPS. As shown earlier, the reading speed increased with increasing school grade.

### Critical print size

The median CPS for the children in all the grades was 0.0 logMAR (IQR = 0.1). The Kruskal–Wallis test failed to reveal statistically significant differences in CPS between grades (*p* = 0.074).

### Reading acuity

**Fig. 6:**
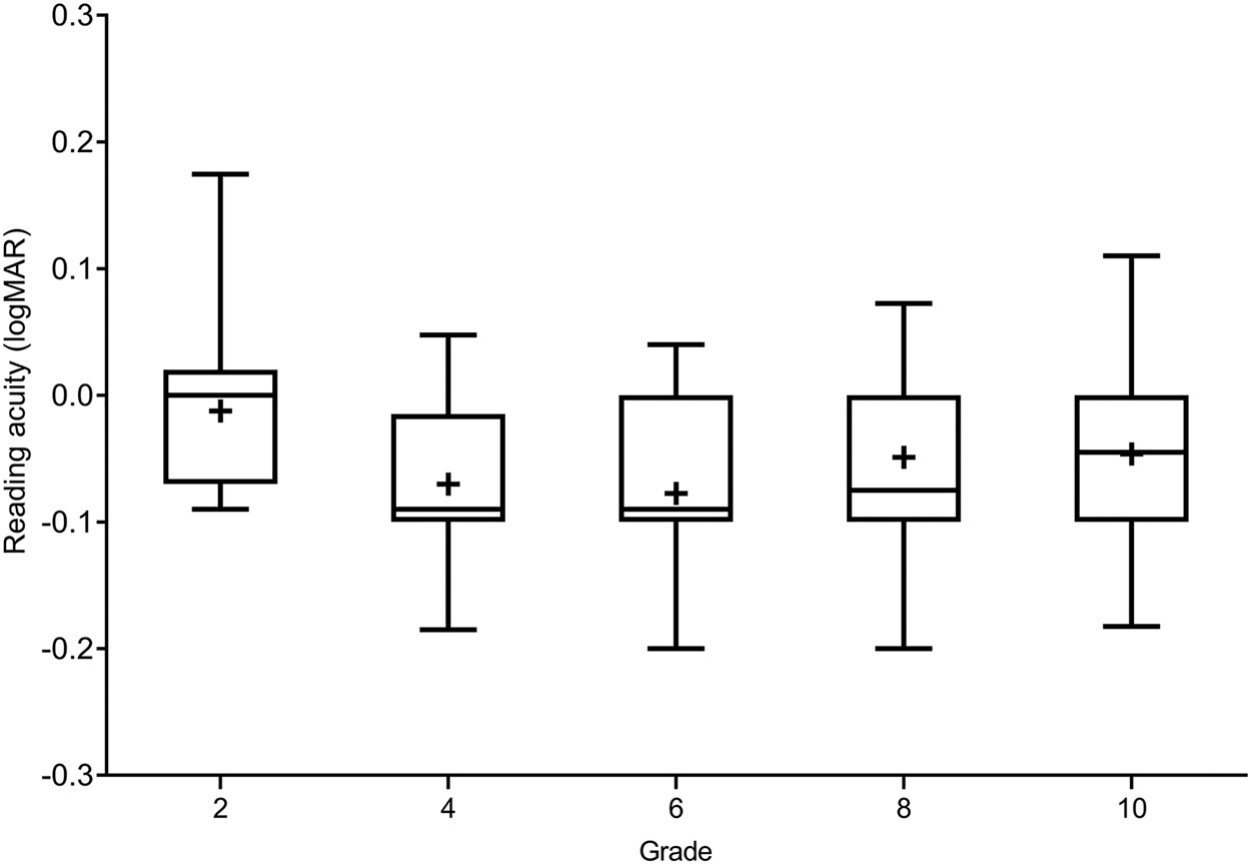
Box and whisker plot showing RA for each school grade. Boxes show 25th to 75th percentile and whiskers range from 5th to 95th percentile, a “+” sign indicates the mean, and the horizontal line represents the median.

The median RA for the 2nd grade was 0.0 logMAR (IQR = 0.09), 4th grade was −0.09 logMAR (IQR = 0.09), 6th grade was −0.09 logMAR (IQR = 0.1), 8th grade was −0.08 logMAR (IQR = 0.1) and 10th grade was 0.05 logMAR (IQR = 0.1). The Kruskal–Wallis test revealed that the differences in RA between school grades were statistically significant, *H*(4) = 14.62 (*p* < 0.01). A post hoc Dunn multiple comparison revealed that RA for the 2nd grade was different from RA for the 4th grade (*p* = 0.015) and for the 6th grade (*p* = 0.004). Fig. 6 shows the box and whisker plot for RA across the grades.

## Discussion

This study reports normative reading performance for young children for the Portuguese MNREAD reading acuity chart. These results are relevant for the purpose of screening reading disabilities and as a clinical reference for prescribing low vision aids in children with impaired vision. Our main findings are that the maximum reading speed increased and reading accessibility index increased with increasing age and school grade. Reading acuity shows initial improvement from early school years and gradually stabilizes in more mature children. We failed to find significant changes in CPS between school grades.

The maximum reading speeds in our study were similar to those of Virgili et al.^8^ in Italian children for the 4th, 6th, and 8th grades. Reading speed for 2nd grade children was comparatively lower in our study. The difference might be because the Portuguese MNREAD chart has been designed with the help of children in the 3rd grade in Brazil. Therefore text might be a bit difficult for children in the 2nd grade tested in the present study.^6^ Another important aspect is that the Portuguese version of the MNREAD chart was designed and validated using Brazilian Portuguese and subtle differences in the linguistics might have also impaired the reading speed in the 2nd grade children.

Reading speed of 2nd grade children for the Portuguese version of the MNREAD test was 57 wpm compared to 137 wpm reported by Calabrèse et al.^11^ in the English version of the MNREAD chart. Nevertheless, the rapid increase of reading speed in Portuguese children resulted in a plateau of around 180 wpm for 16-year-old children to a plateau of 202 wpm reported in Calabrèse et al.^11^ Both the Italian and Portuguese version of the MNREAD chart yield similar reading speed, which was 10 to 20 wpm slower than the reading speed reported previously using the English version of the MNREAD chart.^8^^,^^11^^,^^18^ Other studies using the IReST reading test have also observed differences in reading speed between English and Portuguese readers. The mean difference in IReST reading test between English and Portuguese adult readers was approximately 20 wpm.^19^ The dissimilarities in reading speed between the Portuguese and English children were within the anticipated differences. With age and learning children's reading performance evolves to be more proficient, which gives a better comprehension and a faster reading rate.^20^^,^^21^ This is consistent with our results that the participants are reading at a faster pace with increasing grades.

In the current study the rate of growth in reading speed was 14.5 wpm/year (0.057 log wpm/ year). This finding is in line with the previous studies by Virgili et al.^8^ and DeCarlo et al.^18^ Virgili et al.^8^ reported an increase of 0.052 log wpm per grade in Italian children (approximately 13 wpm/year) while DeCarlo et al.^18^ reported an increase of 10.6 wpm/grade (confidence interval between 6.2 and 15.0 wpm/grade) in their control group of normal sighted children for the version of the English MNREAD chart. Calabrèse et al.^11^ found slower increase in reading speed than in the current study, with approximately 8.13 wpm/year.

We failed to find any significant difference in critical print size across grades and this finding was in agreement with Calabrèse et al.^11^ However, Virgili et al.^8^ reported a linear increase of CPS with increasing grade, but the CPS was similar for all grades except 5th and 8th grade. One possible explanation for this is the NLME model with 80% MRS cutoff that could have resulted in CPS similar for all the grades. Also, CPS is recorded in discrete steps of 0.1 logMAR which is a larger approximation than other parameters leading to a range effect for these children whose vision are in the narrow normal range.

Our results indicated significant improvements in reading acuity from 2nd to 4th grade (0.0 logMAR to −0.09 logMAR) and remained stable for all other grades. This finding is in line with Virgili et al.^8^ who also reported the RA became better with increasing age. This contrasts with Calabrèse et al.,^11^ who found a steeper slope of improvement from 8-year-old to 16-year-old (−0.10 logMAR to −0.18 logMAR). Our results and Virgili et al.^8^ results show that reading acuity is considerably worse in Italian (0.06 logMAR) and Portuguese (0.00 logMAR) children than in children reading the English MNREAD acuity chart (−0.10 logMAR). This could be due to the presence of diacritical marks in the Italian and Portuguese languages that might lead to more crowding phenomenon than English language especially given the small print size and inexperienced young readers.

One limitation of this study is that refraction was not performed for the participants. While efforts were made to ensure that the visual acuity of the children was close to normal, not accounting for potential refractive errors that could introduce variability in the results. Therefore, the impact of uncorrected refractive errors on the reading parameters should be considered when interpreting the study findings.

In conclusion, we have provided normative reading performance data for the Portuguese version of the MNREAD reading acuity test. This information is useful for clinicians to prescribe optimum magnification for children with low vision and for schoolteachers to identify children with reading disabilities in the Portuguese speaking population. This would also help researchers to adopt reading performance as an outcome measure in clinical research.

## Conflicts of interest

The author has no conflicts of interest to declare.

## References

[1] Macedo AF, Crossland MD, Rubin GS. (2011). Investigating unstable fixation in patients with macular disease. Invest Ophthalmol Vis Sci.

[2] Miranda AM, Nunes-Pereira EJ, Baskaran K, Macedo AF. (2018). Eye movements, convergence distance and pupil-size when reading from smartphone, computer, print and tablet. Scand J Optometr Visual Sci.

[3] Brussee T, van Nispen RM, van Rens GH. (2014). Measurement properties of continuous text reading performance tests. Ophthalmic Physiol Opt.

[4] Hernandez-Moreno L, Senra H, Marques AP, Perdomo NM, Macedo AF. (2023). The basic VRS-effect study: clinical trial outcomes and cost-effectiveness of low vision rehabilitation in Portugal. Ophthalmol Ther.

[5] Legge GE, Ross JA, Luebker A, LaMay JM. (1989). Psychophysics of reading. VIII. The Minnesota low-vision reading test. Optom Vis Sci.

[6] Castro CT, Kallie CS, Salomão SR. (2005). Development and validation of the MNREAD reading acuity chart in Portuguese. Arq Brasil Oftalmol.

[7] Baskaran K, Macedo AF, He Y (2019). Scoring reading parameters: an inter-rater reliability study using the MNREAD chart. PLoS One.

[8] Virgili G, Cordaro C, Bigoni A, Crovato S, Cecchini P, Menchini U. (2004). Reading acuity in children: evaluation and reliability using MNREAD charts. Invest Ophthalmol Vis Sci.

[9] Mataftsi A, Bourtoulamaiou A, Haidich AB (2013). Development and validation of the Greek version of the MNREAD acuity chart. Clin Exp Optom.

[10] Idil SA, Caliskan D, Idil NB. (2011). Development and validation of the Turkish version of the MNREAD visual acuity charts. Turk J Med Sci.

[11] Calabrese A, Cheong AM, Cheung SH (2016). Baseline MNREAD measures for normally sighted subjects from childhood to old age. Invest Ophthalmol Vis Sci.

[12] Hernandez-Moreno L, Senra H, Lewis P (2020). Cost-effectiveness of basic vision rehabilitation (the basic VRS-effect study): study protocol for a randomised controlled trial. Ophthalmic Physiol Opt.

[13] Camparini M, Cassinari P, Ferrigno L, Macaluso C. (2001). ETDRS-fast: implementing psychophysical adaptive methods to standardized visual acuity measurement with ETDRS charts. Invest Ophthalmol Vis Sci.

[14] Core Team R (2022).

[15] Calabrèse A, Mansfield JS, Legge GE. mnreadR, an R package to analyze MNREAD data. 2018.

[16] Cheung SH, Kallie CS, Legge GE, Cheong AM. (2008). Nonlinear mixed-effects modeling of MNREAD data. Invest Ophthalmol Vis Sci.

[17] Calabrese A, Owsley C, McGwin G, Legge GE. (2016). Development of a reading accessibility index using the MNREAD acuity chart. JAMA Ophthalmol.

[18] DeCarlo DK, Gao L, McGwin G, Owsley C, Kwon M. (2020). Repeatability and validity of MNREAD test in children with vision impairment. Transl Vis Sci Technol.

[19] Trauzettel-Klosinski S, Dietz K, Grp IRS. (2012). Standardized assessment of reading performance: the new international reading speed texts IReST. Invest Ophthalmol Vis Sci.

[20] Carver RP. (1992). Reading rate - theory, research, and practical implications. J Read.

[21] Morris D, Bloodgood JW, Perney J (2011). Validating craft knowledge: an empirical examination of elementary-grade students' performance on an informal reading assessment. Elem Sch J.

